# Physical Activity and Type 2 Diabetes: In Search of a Personalized Approach to Improving β-Cell Function

**DOI:** 10.3390/nu15194202

**Published:** 2023-09-28

**Authors:** Emanuela Di Murro, Gianfranco Di Giuseppe, Laura Soldovieri, Simona Moffa, Ilaria Improta, Umberto Capece, Enrico Celestino Nista, Francesca Cinti, Gea Ciccarelli, Michela Brunetti, Antonio Gasbarrini, Alfredo Pontecorvi, Andrea Giaccari, Teresa Mezza

**Affiliations:** 1 Endocrinologia e Diabetologia, Fondazione Policlinico Universitario Agostino Gemelli IRCCS, 00168 Rome, Italy; elamanudm@gmail.com (E.D.M.); digiuseppegianfranco@gmail.com (G.D.G.); laura.soldovieri@gmail.com (L.S.); simona.moffa@gmail.com (S.M.); nutrizionistailariaimprota@gmail.com (I.I.); capeceumberto@gmail.com (U.C.); cinti_francesca@hotmail.com (F.C.); ciccarelligea@gmail.com (G.C.); brunettimichela2@gmail.com (M.B.); alfredo.pontecorvi@unicatt.it (A.P.); 2 Dipartimento di Medicina e Chirurgia Traslazionale, Università Cattolica del Sacro Cuore, 00168 Rome, Italy; enricocelestino.nista@policlinicogemelli.it (E.C.N.); antonio.gasbarrini@policlinicogemelli.it (A.G.); 3 Pancreas Unit, CEMAD Centro Malattie dell’Apparato Digerente, Medicina Interna e Gastroenterologia, Fondazione Policlinico Universitario Agostino Gemelli IRCCS, 00168 Rome, Italy

**Keywords:** type 2 diabetes, physical activity, exercise, β-cell function, insulin resistance

## Abstract

Type 2 diabetes mellitus (T2DM) is one of the most widespread diseases worldwide. Lifestyle interventions, including diet and physical activity (PA), are fundamental non-pharmacological components of T2DM therapy. Exercise interventions are strongly recommended for people with or at risk of developing or already with overt diabetes, but adherence to PA guidelines in this population is still challenging. Furthermore, the heterogeneity of T2DM patients, driven by differing residual β-cell functionality, as well as the possibility of practicing different types and intensities of PA, has led to the need to develop tailored exercise and training plans. Investigations on blood glucose variation in response to exercise could help to clarify why individuals do not respond in the same way to PA, and to guide the prescription of personalized treatments. The aim of this review is to offer an updated overview of the current evidence on the effects of different regimens and modalities of PA regarding glucose sensing and β-cell secretory dynamics in individuals with prediabetes or T2DM, with a special focus on β-cell function.

## 1. Introduction

T2DM is a chronic metabolic disorder characterized by high glucose levels, driven by the disruption of the physiologic balance between tissue sensitivity to insulin action, changes in plasma glucose and insulin secretion, β-cell function, and mass. In particular, impairments in β-cell function [1,2] can affect the dynamics of insulin secretion even at a very early stage of the disease [3,4]. The increased morbidity and mortality in T2DM patients [5] can be mostly ascribed to metabolic dysregulation and chronic inflammation leading to atherosclerotic cardiovascular disease (ASCVD). Lifestyle interventions, including appropriate diet and physical activity (PA), constitute an important non-pharmacological aspect of T2DM therapy, and their effectiveness in the prevention of T2DM-associated ASCVD has been amply demonstrated [6]. PA improves glycemic control [7] in patients with diabetes by enhancing insulin secretion and insulin sensitivity [8,9], and by stimulating glucose uptake by skeletal muscle and reducing body weight against a background of poor β-cell function. In addition, PA may counter inflammation related to pancreatic β-cell dysfunction [10] via circulating mediators, reducing oxidative stress and increasing the levels of antioxidants [11,12,13]. In this regard, some meta-analyses have shown that aerobic training, resistance training, and the combination of the two improve glycemic control and HbA1c levels [14,15,16], together with significant improvements in anthropometric, cardiovascular, and metabolic risk factors [16,17]. Other studies have found associations between PA and diabetes remission rate in newly diagnosed subjects [18,19,20]. Anyway, it should be underlined that improvements in glucose tolerance and glucose homeostasis were only achieved through PA in combination with pharmacologic treatment and/or an appropriate dietary regimen in these studies, providing evidence that PA is a necessary, but not sufficient, strategy for achieving partial or complete remission from the disease.

The European Society of Cardiology (ESC) Task Force on sport cardiology and exercise has suggested that adults with diabetes should exercise for at least 5–7 days/week with 30 min/day of moderate or vigorous aerobic activity together with 15 min of resistance training. This should preferably be spread throughout the week, with no more than two consecutive days of inactivity, to achieve the best impact on glucose homeostasis [21] and on cardiorespiratory fitness and body composition, with major changes in body fat mass [22]. In addition, the recently published update to the Position Statement on Exercise and T2DM by the American College of Sports Medicine (ACSM) and the ADA [23] has summarized new evidence on the different modalities and intensities of PA, underlining that a “one-size-fits-all” exercise prescription based only on aerobic activity and the possible association of resistance activity is no longer feasible. Although the metabolic benefits of exercise are relevant, its effects are short-lived and begin to fade within 48 h [21,24]; thus, the need for a strategy to maintain the metabolic setting achieved through exercise is evident. Moreover, there is still controversy as to the type of PA that is most effective in controlling glucose metabolism.

The aim of this review is to summarize the current evidence on different types of PA and their effects on β-cell function and metabolic control in individuals with prediabetes or overt diabetes, considering that the beneficial effect of exercise includes improvements in tissue sensitivity to glucose and insulin, as well as insulin content and/or insulin secretion. Specifically, we will focus on the interrelationship between PA and changes in β-cell function.

## 2. Evidence Acquisition

The literature search was performed, in duplicate and independently, by two authors (EDM and GDG) through the PubMed database, focusing on exercise intervention in individuals with type 2 diabetes. The initial search included the following MeSH term combination: diabetes mellitus type 2, physical activity, exercise, β-cell function, hyperglycemia, training, and metabolic dysfunction. Titles and abstracts from the literature search were independently evaluated by three investigators (EDM, GDG, and TM). Only eligible full texts in the English language, like scientific papers, clinical trials, meta-analyses, and reviews published up to August 2023, were considered. Case reports and series were excluded. The literature search was limited to human studies.

## 3. Effects of Physical Activity on β-Cell Function and Insulin Secretion

Insulin resistance and β-cell dysfunction are fundamental metabolic defects of T2DM, and these conditions are already partially present in obese subjects with prediabetes [25]. The preservation of residual β-cell function in individuals with obesity and IGT—who have already lost up to 50–70% of β-cell function—is an effective strategy to prevent T2DM [26,27]. The mechanism linking PA and β-cell function is still not fully understood; however, it has been demonstrated that exercise interventions and correct dietary management may protect β-cell function by reducing metabolic stress [28]. Exercise training has a potential role in preserving β-cell health, and, therefore, represents an effective strategy to prevent or delay diabetes onset in individuals at risk of or already affected by the disease; moreover, PA supports pharmacological effectiveness to prevent β-cell failure [10].

It can be difficult to estimate the relative contribution of a single type of PA to β-cell protection, as well as the correct timing for exercise interventions in patients with metabolic impairments, but there is evidence that exercise, when started at an early stage, may delay disease progression by increasing β-cell resistance to endoplasmic reticulum (ER) stress [29] and other mediators of immune damage [10]. Several trials have studied the effect of exercise on insulin secretion in individuals with T2DM and prediabetes [8,9,30,31,32,33,34,35,36,37], as well as in sedentary overweight or obese subjects [8,35,36,38,39,40,41]. Some of these studies have investigated the effects of different intensities of aerobic training [8,9,30,32,34,35,36,37,40], while others have evaluated high-intensity interval training (HIIT) [42,43], resistance training [31,36], and simultaneous aerobic and resistance training or a combination of the two [38,39,41] on metabolic outcomes (Table 1). Slentz et al. [40], in the STRRIDE project, evaluated the effects of different intensities and different amounts of exercise on the Disposition Index (DI) in an 8-month intervention program in people with T2DM. The DI represents the product of measures of insulin sensitivity and the acute insulin response to glucose (AIR_g_, an index of first-phase insulin secretion), thus expressing the ability of β-cells to compensate for augmented insulin resistance with changes in the first-phase secretion [44]. This index has been shown to decrease with the deterioration of glucose tolerance, as well as to predict diabetes onset independently of fasting and 2 h plasma glucose levels after an oral glucose tolerance test (OGTT) [45,46]. The STRRIDE study demonstrated that moderate and vigorous intensity exercise improved β-cell function through distinct mechanisms in sedentary overweight adults. A high amount of exercise and exercise of vigorous intensity were associated with an improvement in insulin sensitivity and a compensatory decrease in insulin secretion, while a low amount of exercise and moderate-intensity exercise were associated only with a comparable improvement in insulin sensitivity. Moreover, moderate exercise led to greater improvements in β-cell function, while vigorous exercise was superior in terms of achieving improvements in cardiorespiratory fitness. Further, to investigate the effects of aerobics, resistance training, and their combination on insulin sensitivity and insulin secretion, an intravenous glucose tolerance test (IVGTT) was performed in the STRRIDE cohort [41] at baseline, at the end of exercise training (16–24 h after the last exercise) and 14 days after the end of exercise training. This analysis demonstrated that the combination of aerobic (AT) and resistance training (RT) led to greater improvements in insulin sensitivity, DI, and glucose effectiveness compared to AT or RT alone. In particular, a greater improvement in β-cell function was found in people with T2DM compared to normal, glucose-tolerant (NGT) subjects [47].

In conclusion, in the cited trials, it is important to note that only interventions lasting more than 2 months resulted in improvements in insulin secretion, and that all training regimens led to benefits regarding insulin secretion. This emphasizes that significant results can only be achieved following an appropriate exercise program, whereas random exercise sessions might not result in significant improvements in glucose metabolism or cardiovascular outcomes.

## 4. Responsiveness to Physical Activity

Despite the well-recognized positive impact of PA on β-cell function and insulin secretion, individuals with different secretory functions at baseline may show different responses to interventions, and a variable proportion of patients have been described as exercise resistant in several studies. Dela et al. [9] stratified patients with T2DM into “moderate” and “low” secretors according to individual C-peptide responses to an intravenous glucagon test. They found that only moderate secretors obtained positive responses from an aerobic training program, thus highlighting that a residual β-cell secretory capacity is required in order to maximize the beneficial effect of aerobic exercise on glycemic control. In addition, to further investigate how baseline β-cell function may influence training-induced changes in glycemic control, the HERITAGE study [34] assessed insulin secretion patterns during an IVGTT in a large heterogenic population to detect possible differences in β-cell functional effects in response to a 20-week endurance training program (cycle ergometers 3 days/week for 60 sessions). Participants with better glucose tolerance at baseline showed reduced insulin secretion following regular exercise, while those with poorer glucose tolerance at baseline showed an increase in insulin secretion after exercise. These results suggest that intersubject variability in baseline β-cell function could partially explain the different effects of exercise on glucose homeostasis. In this context, intersubject variability in basal insulin secretion could provide an effective explanation for the different secretory responses to exercise and should be considered when prescribing tailored and individualized treatments. Consistently, another study confirmed this variability by demonstrating that long-term exposure to hyperglycemia –leading to reduced β-cell function [48] and low insulin secretory function—before the introduction of PA predict a poorer training-induced outcome. Thus, aerobic exercise may be less effective in improving glucose control in patients with poorly controlled long-term T2DM. Accordingly, another study [36] confirmed that the response to exercise in prediabetic and diabetic subjects largely depends on basal β-cell function: in particular, basal HbA1c, BMI, and β-cell function were all predictors of exercise response in elderly patients with prediabetes; by contrast, the type of PA did not predict the effectiveness of the response. In this context, residual β-cell function should be restored through appropriate pharmacological treatment by eliminating the noxious effects of gluco- and lipotoxicity before therapeutic aerobic exercise can provide effective improvements in glucose homeostasis. Collectively, these data strongly support the need for individualized treatments in order to maximize the benefit of exercise.

A recent study [18] investigated the effectiveness of a 12- or 24-month intensive lifestyle program in inducing partial or complete T2DM remission compared to standard of care, demonstrating that 23% of participants in the lifestyle intervention group met the criteria for any T2DM remission compared to 7% in the standard care group. Further, the highest remission rate was obtained in participants with well-controlled short-duration T2DM. Similarly, a 6-month exercise and weight loss program, which included 45–60 min sessions/week of supervised on-site exercise and home walking on other days for a total of 5–6 days per week of exercise in newly diagnosed and drug-naïve T2DM individuals, [19] induced partial remission in 80% of subjects who completed the program.

In the LookAHEAD study [20], individuals with a median T2DM duration of 5 years were randomized to an intensive lifestyle-based weight loss intervention (ILI) aimed at reducing total caloric intake by limiting total and saturated fat intake and increasing PA up to 175 min/week, or a Diabetes Support and Education intervention (DSE). The results showed that time since diagnosis, baseline HbA1c, and amount of weight loss were independent predictors of T2DM remission. Moreover, in the PLIS study [49], 908 individuals with prediabetes were stratified into low-risk or high-risk phenotypes based on insulin secretion, insulin resistance, and liver fat content. Low-risk patients were randomly assigned to conventional lifestyle intervention (eight lifestyle intervention sessions spread out over one year, in which they were advised to perform 3 h of exercise weekly) or no lifestyle intervention. On the other hand, high-risk individuals were assigned to conventional or intensified lifestyle interventions, with double the amount of exercise, i.e., 16 one-to-one coaching sessions. Over the 3-year follow-up period, the intensified lifestyle intervention in high-risk individuals proved to be more effective in normalizing glucose tolerance.

Collectively, these results confirm the importance and the effectiveness of exercise as an early treatment for T2DM patients. Moreover, the exercise prescription needs to be personalized, aiming at diabetes prevention or remission on the basis of risk phenotype. Further investigations on the effectiveness of different types of exercise on β-cell function in the heterogeneous T2DM population are therefore necessary.

## 5. Exercise Regimens for T2DM

As shown in Table 2, many types of PA can improve glycemic control and lead to overall benefits in people with T2DM. To date, aerobic and resistance training, and the combination of both, have formed the cornerstone of lifestyle intervention for T2DM patients, as previously mentioned. Aerobic training, like running and cycling for about 1 h, is a moderate-intensity type of PA and represents the best activity to reduce body fat mass when required. Moreover, it leads to improvements in oxygen transport and metabolism in the skeletal and heart muscle [50]. Resistance training, on the other hand, is strength-building, as it increases lean body mass, and is characterized by short-term exercise repetitions performed by using, for example, external resistance tools like dumbbells, kettlebells, and barbells, or using body weight, weight machines, or elastic resistance bands.

Many studies have investigated the effects of different training regimens and their combinations on glucose homeostasis, lipid profile, blood pressure, and other metabolic and fitness parameters [51], as well as their anti-inflammatory activity [52] in prediabetic [53] and diabetic [22,54,55] patients, with or without weight loss. Among these, the HART-D [22] and DARE [56] trials have aimed at studying possible changes in HbA1c levels resulting from aerobic training, resistance training, and the combination of both in T2DM adults. The DARE study demonstrated significant improvements in glycemic control in the whole population, with a greater reduction in HbA1c in the combined exercise group compared to resistance and aerobic training alone, and these effects were greater in individuals with poor glycemic control at baseline. However, since the study design envisaged longer exercise durations for the combination group compared to the other two groups, this could partially influence the improvements observed in the combined exercise group. In contrast, the training programs in HART-D were designed to maintain similar weekly training times among groups, and the improvements in HbA1c levels were lower in the DARE population. This could, however, be due to the longer diabetes duration in HART-D trial participants, with 18.3% of the subjects on active insulin treatment (while insulin treatment was an exclusion criterion in DARE), or to the higher female population. Moreover, Cuff et al. [57], in a small population of 28 postmenopausal, obese, and diabetic women, randomized them to three exercise types (aerobic, resistance, or combination training) for 4 months and demonstrated that combined training elicited significant enhancement in insulin sensitivity compared to the control group. It seemed to be more successful in improving insulin resistance than aerobic training alone, while no changes in HbA1c levels were observed, probably due to the small cohort which was enrolled.

Indeed, in a systematic review by Schwingshackl et al. [58], aerobic and combined training were found to be more effective in reducing HbA1c compared to resistance training alone. In particular, the combined training produced a significantly greater reduction in HbA1c and fasting glucose, as well as improvements in lipid and blood pressure profiles compared to aerobic or resistance training alone.

Despite the major effects of improving joint range of motion; reducing the risk of falls, especially in adults with peripheral neuropathy; and facilitating participation in other activities, trials investigating the effects of flexibility and balance exercises, alone or in combination with resistance training, showed no significant impact on glucose control [59,60,61]. In addition, Pilates and yoga [62,63,64], considered alternative training regimens for individuals with lower levels of fitness and poorer balance, also demonstrated improvements in postprandial glycemia after 4 weeks and in HbA1c levels after 12 weeks of treatment in older women with T2DM who underwent 12 weeks of Pilates training (60 min per session/3 times per week). Similarly, two recent meta-analyses reviewed the efficacy of yoga in adults with T2DM [63,64], concluding that yoga significantly improves glycemic outcomes such as fasting and postprandial blood glucose as well as Hb1Ac levels, lipid profile, and blood pressure.

Recently, high-intensity interval training (HIIT) has been developed as a new PA protocol. It consists of the repetition of short intervals of aerobic training (running or cycling), performed between 75–95% of peak heart rate, alternating with short periods of active or passive recovery. In a recent metanalysis, Jelleyman et al. [65] aimed to quantify the effect of HIIT on markers of glucose regulation and insulin resistance compared with controls or continuous training, and concluded that participants in HIIT groups had a 0.19% reduction in HbA1c and a 1.3 kg reduction in body weight compared to control groups with interventions lasting at least 2 weeks. However, significant intrasubject heterogeneity to exercise responsiveness in terms of glycemic control and β-cell function was observed in a group of T2DM patients after 8 weeks of low-volume HIIT, thus stressing the need to personalize exercise treatments. Similarly, functional high-intensity training (F-HIT) is a supervised training protocol of 10–20 min/session 3 times a week, characterized by various functional movements (based on real-world situational exercises) at a high intensity, combining resistance training, gymnastics (body weight), and aerobic exercise. F-HIT was found not only to be a time-efficient modality with which to improve fitness levels, insulin sensitivity, and pancreatic β-cell function in adults with T2DM [43], but also to reduce the risk of metabolic syndrome [66]. Fealy et al. [67] recently demonstrated the benefits of this activity protocol on β-cell function in people with T2DM, showing that F-HIT is effective in improving insulin sensitivity and reducing cardiometabolic risk in overweight or obese adults with T2DM. In particular, F-HIT exercise led to reductions in fat mass, plasma triglycerides and cholesterol, metabolic syndrome severity, diastolic blood pressure, and concentrations of plasma resistin—a pro-inflammatory adipokine. A recent review has indeed highlighted how only high-intensity physical exercise improves metabolic control at the musculoskeletal level, possible due to the release of myokines, fatty acids, and/or the activation of specific β-cellular receptors for fatty acids [68]. In addition, another study investigating the effects on beta-cell function suggested that F-HIT significantly increased the DI, while no changes in the OGTT-derived first- and second-phase insulin secretion were observed after intervention [43]. In summary, compared to other types of exercise, and with a minimal time commitment, F-HIT and HIIT can be considered as safe and effective training approaches, leading to improvements in β-cell function in adults with T2DM with preserved residual β-cell secretory capacity and with no contraindications.

## 6. Adherence and the “Lack of Time”

Currently, there are many lifestyle-related obstacles to the achievement of the minimum recommended PA levels. One of the main barriers to adherence is “lack of time” [69]. Guidelines advocate at least 5 h of aerobic, resistance, and flexibility training spread out over 5 days per week, but compliance and adherence to these recommendations is very low; in fact, only 10–23% of people adhere to these recommendations in the USA [70]. In addition, the estimation of PA, using a structured 7-day physical activity recall interview in medically treated T2DM individuals with atherosclerotic cardiovascular disease or with cardiovascular risk factors, revealed that fitness levels were very low and almost half the participants failed to reach the minimum PA recommendation levels [71]. In this context, “lack of time” represents one of the main reasons for non-adherence to therapeutic exercise programs. Thus, additional efforts are needed in order to promote greater adherence to guidelines by prescribing tailored PA programs. In addition, it should be considered that the extent of the beneficial effects—such as the improvement of glucose homeostasis and the reduction in cardiometabolic risk factors in people with diabetes—is dependent on the total energy expenditure of training rather than exercise duration or intensity [72,73]. In particular, the American College of Sports Medicine (ACSM) recommends that T2DM patients undergo at least 1000 kcal/week of energy expenditure and that men and women expend between 1000 and 2000 kcal/week to obtain cardiometabolic benefits [23]. Therefore, new PA high-intensity exercise protocols (e.g., HIIT and F-HIT) have been studied as possible alternatives with which to improve adherence in people with a “lack of time”. High-intensity exercise protocols have been proposed as time-efficient methods to achieve cardio-metabolic health outcomes, equivalent to traditional aerobic training programs [74]. In addition, HIIT could be an effective protocol to use to meet the 1000–2000 kcal/week expenditure goal. In this regard, a study [37] investigated the relationship between exercise dose and pancreatic β-cell function in men and women with prediabetes. The exercise intervention consisted of a supervised aerobic exercise program of 50–60 min 5 days/week at 60–65% of the maximum heart rate (HR) for the first 4 weeks and, thereafter, the exercise intensity was increased and maintained at 80–85% HR. Moreover, the subjects also followed a controlled dietary regimen. The study showed that increasing the intensity of exercise can enhance β-cell function in adults with poor insulin secretion capacity, without any relationship to changes in body fat. In particular, the study showed that exercise interventions expending more than 2000 kcal/week increased pancreatic β-cell function in a linear dose–response manner and confirmed that a tailored exercise prescription with the correct intensity, duration, and modality is necessary to maximize energy expenditure and glucoregulatory effects. Similarly, Davis et al. [75] reported that that high-dose exercise, compared to low-dose exercise, enhanced first-phase β-cell function in overweight children.

To summarize, high-intensity exercise programs could be effective in reaching energy expenditure targets within the shortest possible time, thus increasing the rate of adherence to recommendations. However, this PA protocol should be prescribed only after a cardiovascular screening test—including resting ECG and maximal stress ECG testing—and only after accurate anamnesis, excluding congenital heart disease, ischemic heart disease, myocarditis, and arrhythmias, as well as any pathologic bone, joint, or muscle condition.

Among cardiovascular screening tests, cardiopulmonary exercise testing (CPET) is a non-invasive yet valuable screening tool that provides a comprehensive and multi-parametric assessment of the functioning of the cardiovascular, pulmonary, muscular, and even cellular systems during exercise. Several studies employing this type of test have highlighted specific cardiorespiratory alterations in patients with T2DM and without any overt pulmonary or cardiovascular disease. These alterations include reduced peak workload, peak oxygen uptake, oxygen pulse, and ventilatory efficiency [76].

Moreover, CPET has facilitated the identification of significant gender differences [77] in a fundamental parameter derived from the test, namely, peak oxygen uptake (VO2peak), which reflects functional capacity and cardio-circulatory efficiency. In the context of tailoring exercise prescriptions, it is essential to consider not only that a diabetic patient may exhibit a lower VO2peak value compared to a non-diabetic individual, but also that sedentary women with diabetes have a lower VO2peak compared to men with the same diagnosis.

## 7. Nutrition and Physical Activity

Together with PA, nutrition is another fundamental non-pharmacological milestone in T2DM therapy. Over the last years, many recommendations [78,79,80,81] have been put forth by scientific communities to help clinicians in the prescription of the most appropriate nutritional intervention in subjects with T2DM: in fact, by promoting weight loss and ameliorating insulin sensitivity, diet and PA together can lead to improved blood glucose and blood pressure profiles, as well lipid levels, with effectiveness for CV safety [82]. However, adherence in clinical practice seems to be generally poor [25], and more effort should be put in building combined and tailored nutritional/exercise plans to improve overall compliance. The study of food metabolism is complex, and its consequences on overall health depend on the interrelationships among nutrients and bioactive non-nutrients (antioxidants, fiber, minerals, etc.) rather than single nutrients alone; for these reasons, in recent decades, more emphasis has been put on the quality of one’s diet [83] and possible dietary patterns according to individual goals. The Mediterranean diet deserves special mention: it is characterized by the consumption of whole grain, legumes, fruits, vegetables, nuts, olive oil, and moderate intake of wine and meat, providing an important apport of vitamins, minerals, anti-oxidants, mono- and poly-unsaturated fatty acids, and fibers. It has recently been demonstrated to correlate with more favorable cardiovascular risk factors profile, better glucose control, and lower BMI [84].

In accordance with the 2020–2025 US Dietary Guidelines for Americans, a healthy eating plan should provide the appropriate daily caloric intake. It should emphasize the consumption of fruits, vegetables, and whole grains; include reduced or non-fat dairy products, lean meats, poultry, fish, beans, eggs, and nuts; and keep saturated and trans fats, cholesterol, salt, and added sugar intake low. A whole foods-based diet is rich in micronutrients and antioxidants, and is beneficial for preventing and managing T2DM [85]. In particular, a nutritional program for patients with T2DM undergoing an exercise regimen should be carefully planned to improve blood glucose levels and promote overall health. Typically, individuals with T2DM who engage in regular physical activity should focus on maintaining a proper carbohydrate balance by limiting foods with high glycemic indices. They should also incorporate sources of lean protein and fiber to enhance glycemic control, while opting for unsaturated fats.

Among all dietary approaches, carbohydrate restriction has been shown to reduce body weight and improve glycemia [25], and the use of popular diet options (i.e., low carbohydrate, ketogenic diet) and other eating patterns (i.e., Mediterranean, vegan) are frequently followed for T2DM management [86]. For example, a pilot study demonstrated the effectiveness of a very low-carbohydrate ketogenic diet in improving glucose control and weight in a group of overweight T2DM subjects compared to a standard low-fat diet [87]. Further strategies include intermittent fasting (IF) and fasting-mimicking diets (FMD) [88]. IF provides alternating periods of eating and fasting and time-restricted feeding, and is an effective method for weight and calorie intake management; FMD is a plant-based dietary regimen designed to obtain fasting-like metabolic effects. Both IF and FMD offer potential advantages, including weight loss and enhanced metabolic markers [25]. Furthermore, we have recently reviewed possible dietary strategies (including carbohydrate and glycemic index restriction, whey proteins, and meal timing) which lead to positive effects on β-cell function [3].

Recently, a potential correlation has been identified between nutrition timing and physical exercise. Specifically, PA performed in a fasted state by healthy individuals leads to greater improvements in insulin sensitivity compared to the same type of exercise performed in a different nutritional state [89]. This research, combined with the findings of a retrospective cohort study on the LookAHEAD population [20], which revealed that greater changes in HbA1c were independent of weekly training volume, but, rather, related to the time of day when physical activity occurred, marks the beginning of an intriguing frontier in scientific research regarding lifestyle interventions and circadian biology. This research has the potential to optimize the effectiveness of both pharmacological and non-pharmacological treatments for patients with type 2 diabetes mellitus [90].

Further investigations are necessary in order to explore the efficacy, compliance, long-term sustainability, metabolomic implications, and potential interactions of various nutritional strategies when combined with different patterns of physical activity on glucose homeostasis and β-cell function. Additionally, these investigations should consider their associations with pharmacological agents.

## 8. Conclusions

Future approaches to lifestyle intervention in patients with T2DM should certainly consider customized prescription of PA. A tailored training plan should be based on accurate phenotyping, which takes into account the type of diabetes, time since diagnosis, age, sex variabilities, and the presence of diabetes-related complications. Moreover, PA regimens should consider intrasubject variabilities driven by different residual β-cell function; in this scenario, the assessment of the disposition index could be an effective way to establish residual β-cell capacity. To date, considerable evidence has shown that the combination of aerobic and resistance training exerts major effects on glucose regulation in T2DM patients, as well as on the prevention of T2DM-associated ASCVD. However, alternative regimens, such as high-intensity exercise protocols, should be considered in selected patients to maximize the time-effectiveness of PA. Additional trials investigating the effects of exercise duration, intensity, and frequency on specific β-cell functional parameters are needed in order to personalize the doses and modes of exercise and lifestyle interventions to maximize the effect of PA on β-cell function and metabolic and cardiovascular outcomes.

## Figures and Tables

**Table 1:** Clinical studies evaluating the effects of different patterns of PA on β-cell function and glucose homeostasis.

**Refs.**	**Year**	**Population**	**Intervention**	**Duration** **(Weeks)**	**Frequency** **(Sessions/Week)**	**Intensity** **(min/Session)**	**Dietary** **Intervention**	**Effects on** **β-Cell Function**	**Additional Effects on** **Glucose Homeostasis**
Solomon [8]	2010	NGT obese subjects	AT	12	5	60	✔️	↓ OGTT-derived IS (not significant when corrected for IR)	-
		T2DM obese subjects	AT	12	5	60	✔️	↑ OGTT-derived IS	-
Dela [9]	2004	T2DM subjects	AT vs. no exercise	12	5	30–40	✕	↑ β-cell secretory capacity in subjects with preserved β-cell function at baseline	-
Bloem [30]	2008	IGT overweight/obese subjects	AT	1	7	60	✕	↓ AIR_g_ ↑ DI	↑ Insulin sensitivity
Croymans [31]	2013	Non-diabetic overweight/obese subjects	RT	12	3	60	✕	↑ DI	↑ Insulin sensitivity (OGTT-derived mISI) ↓ glucose AUC_OGTT_ ↓ insulin AUC_OGTT_
Dela [32]	2010	Normal weight or overweight first-degree relatives of T2DM subjects	AT (endurance)	12	6	45	✕	=glucose-stimulated IS	↑ glucose-mediated GU
		Control group	AT (endurance)	12	6	45	✕	=glucose-stimulated IS	↑ insulin-mediated GU ↑ glucose-mediated GU
Hordern [33]	2008	T2DM overweight/obese subjects	AT + RT	4	3	80–85	✕	=HOMA-β	Baseline glucose and HbA1c were predictors of ↓ blood glucose after intervention
Boulè [34]	2005	Non-diabetic overweight/obese subjects	AT (endurance)	20	3	30–50	✕	↑ AIR_g_ (in the quartile with the worst baseline GT) ↓ AIR_g_ (in the quartile with the better baseline GT)	-
Solomon [35]	2013	IGT and T2DM overweight/obese subjects	AT	12-16	4–5	60	✕	↑ 1st and 2nd-phase DI	-
He [36]	2016	Prediabetic normal weight and overweight subjects	AT vs. RT vs. control	93	3	50–60	✕	↓ HOMA-β (in both groups vs control) Baseline HOMA-β, HbA1c and BMI were predictors of positive β-cell response to training	↓ glucose levels ↓ HbA1c ↓ HOMA-IR (in both groups vs control)
Malin [37]	2013	Prediabetic obese subjects	AT	12	5	60	✔️	↑ 1st and 2nd-phase DI (related to exercise-dose)	-
Bacchi [38]	2012	Overweight T2DM patients	AT + RT	16	3	60	✔️	No major effects	Baseline HbA1c was predictor of changes in HbA1c after intervention
Lee [39]	2012	Non-diabetic obese adolescent subjects	AT vs. RT vs. no-exercise	12	3	60	✕	=IS at hyperglycemic clamp =DI at hyperglycemic clamp	↑ Insulin sensitivity only in RT group =GT
Slentz [40]	2009	Non-diabetic overweight/obese subjects	AT (at different amount/intensity) vs. no exercise	32	Variable	Variable	✕	↑ DI (moderate-intensity group displayed major improvements in the DI)	-
AbouAssi [41]	2015	Non-diabetic overweight/obese subjects	AT vs. RT vs. AT + RT	32	Variable	Variable	✕	↑ DI ↑ glucose effectiveness (in the AT + AR group compared to AT and AR groups alone)	-
Madsen [42]	2015	T2DM overweight/obese subjects	HIIT	8	3	30	✕	↑ DI ↓ HOMA-β	↓ glucose levels ↓ HbA1c ↓ HOMA-IR
		Non-diabetic subjects	HIIT	8	3	30	✕	-	-
Nieuwoudt [43]	2017	T2DM subjects	F-HIT	6	3	10–20	✕	↑ DI =1st and 2nd-phase IS	-

Key and abbreviations: ↑: increased; ↓: decreased; =: no changes; ✔️: yes; ✕: no; AT: aerobic training; RT: resistance training; HIIT: high-intensity interval training; F-HIT: functional high-intensity training; NGT: normal glucose-tolerant; T2DM: type 2 diabetes mellitus; IGT: impaired glucose-tolerant; IS: insulin secretion; OGTT: oral glucose tolerance test; AIR_g_: acute insulin response to glucose; DI: disposition index; mISI: muscle insulin sensitivity index; AUC_OGTT_: OGTT-derived area under the curve; GU: glucose uptake; GT: glucose tolerance; HbA1c: glycosylated hemoglobin; HOMA-IR: homeostatic model assessment for insulin resistance; HOMA- β: homeostatic model assessment for β-cell function.

**Table 2:** Type of physical exercise for adults with T2DM.

**Type of Exercise**	**Description**	**Intensity**	**Frequency**	**Duration**	**Proven Benefits**	**Additional Specification**
Aerobic Training	Rhythmic and repetitive PA that uses large muscle groups, e.g., running, cycling, swimming, dancing, jogging	Moderate (55–74% HRmax) or Vigorous (75–95% HRmax)	5–7 d/week with no more than two consecutive days between bouts	At least 30′/session for ≥150′/week of moderate activity or ≥75′/week of vigorous activity (or an equivalent combination of moderate- and vigorous-intensity, preferably spread throughout the week).	↓ Glycemia with fewer daily hyperglycemic excursions ↓ HbA1c ↑ Insulin sensitivity ↓ Blood lipids ↓ BP ↑ Fitness levels, even without weight loss	-
Resistance Training	Short-term repetition exercises performed using external resistance tools like dumbbells, kettlebells, and barbells, or using body weight, weight machines, or elastic resistance bands	Moderate (50–69% of 1-RM) or Vigorous (70–85% of 1-RM)	2–3 d/week on non-consecutive days	10–15 repetitions/set with 1–3 sets per type of specific exercise	↑ Strength ↑ Bone mineral density ↑ Lean mass ↓ BP ↓ Blood lipids ↑ Insulin sensitivity ↓ HbA1c	-
Pilates	Specific targeted exercises to improve strength, flexibility, and posture, with particular focus on the core	Light	≥2–3 d/week	-	↑ Blood glucose management ↑ Functional capacity	For patients with low levels of fitness and insufficient balance
Yoga	Breath, movement, and meditation to unite mind, body, and spirit	Light	≥2–3 d/week	-	↓ HbA1c ↓ Blood lipids ↑ Improvement body composition	For patients with low levels of fitness and insufficient balance
Balance	Focuses on the ability to maintain proper posture and refers to exercises that are designed to improve and maintain balance	Light	≥2–3 d/week	-	-	↓Risk of falls by improving balance and gait, even in adults with peripheral neuropathy
Flexibility	Stretching and moving a joint through its range of motion	Light	≥2–3 d/week	10″–30″ per stretch (static or dynamic) group; 2–4 repetitions of each	-	↑ Joint range-of-motion; facilitates participation in activities that require flexibility
HIIT F-HIT	HIIT: repetitions of short intervals of vigorous aerobic training (running or cycling) alternating with a short period of active or passive recovery. F-HIT: based on real-world situational exercises at a high intensity by combining resistance training, gymnastics (body weight), and aerobic exercise	Vigorous (75–95% HRmax) follow by active or passive recovery	3 d/week	Repetitions of 10″–4′ of vigorous activity with 12″–5′ of active or passive recovery	↑ Insulin sensitivity ↑ β-cell function in T2DM with preserved residual β-cell secretory capacity ↑ Fitness levels ↓ HbA1c ↓ BMI ↑ CGM	-

Key and abbreviations: ↑: increased; ↓: decreased; HIIT: high-intensity interval training; F-HIT: functional high-intensity training; T2DM: type 2 diabetes mellitus; HbA1c: glycosylated hemoglobin; 1-RM: 1-repetition maximum; HRmax: heart rate max; BMI: body mass index; CGM: continuous glucose monitoring; BP: blood pressure; PA: physical activity.

## Data Availability

No new data were created or analyzed in this study. Data sharing is not applicable to this article.
